# Vacuum-assisted closure therapy in the management of lung abscess

**DOI:** 10.1186/s13019-014-0157-x

**Published:** 2014-09-06

**Authors:** Zsolt Sziklavari, Michael Ried, Hans-Stefan Hofmann

**Affiliations:** Department of Thoracic Surgery, Hospital Barmherzige Brüder Regensburg, Prüfeningerstraße 86, Regensburg, 93049 Germany; Department of Thoracic Surgery, University Regensburg, Franz-Josef-Strauss-Allee 11, Regensburg, 93053 Germany

**Keywords:** Lung infection, Lung abscess, Surgery techniques, Vacuum-assisted closure, Negative-pressure wound therapy

## Abstract

**Background:**

Despite significant advances in the treatment of thoracic infections, complex lung abscess remains a problem in modern thoracic surgery. We describe the novel application of vacuum-assisted closure for the treatment of a lung abscess. The technical details and preliminary results are reported.

**Methods:**

After the initial failed conservative treatment of an abscess, minimally invasive surgical intervention was performed with vacuum-assisted closure. The vacuum sponges were inserted in the abscess cavity at the most proximal point to the pleural surface. The intercostal space of the chest wall above the entering place was secured by a soft tissue retractor. The level of suction was initially set to 100 mm Hg, with a maximum suction of 125 mm Hg. The sponge was changed once on the 3rd postoperative day.

**Results:**

The abscess cavity was rapidly cleaned and decreased in size. The mini-thoracotomy could be closed on the 9th postoperative day. Closure of the cavity was simple, without any short- or long-term treatment failure. This technique reduced the trauma associated with the procedure. The patient was discharged on the 11th postoperative day.

**Conclusions:**

Vacuum-assisted closure systems should be considered for widespread use as an alternative option for the treatment of complicated pulmonary abscess in elderly, debilitated, immunocompromised patients after failed conservative treatment.

**Electronic supplementary material:**

The online version of this article (doi:10.1186/s13019-014-0157-x) contains supplementary material, which is available to authorized users.

## Background

Empyema thoracis is characterised by a preformed layer as a membrane. On the other hand, abscess of the lung is characterized by a pseudomembrane, with poorly defined borders and it will be covered by granulation tissue during the healing of the surrounding parenchyma. Principle of conservative therapy is the adequate drainage; it must be achieved by fiberoptic bronchoscopy or percutaneous catheter (Monaldi's) drainage [1]. Despite significant advances in the treatment of thoracic infections, complex (e.g., failed primary treatment) lung abscess remains a problem in modern thoracic surgery. Large abscesses and anaerobic bacteria are associated with worse outcome [2]. The prognosis is poor in elderly, debilitated, malnourished, and immunocompromised patients. If the patient is medically unstable, a quick evacuation of pus can be performed by thoracocentesis via a chest tube.

Since the introduction of vacuum-assisted closure therapy (VAC therapy), increasing indications for its use in the treatment of acute or chronic wound infections, including pleural empyema, have been reported [3],[4]. Minimally invasive vacuum-assisted closure therapy (Mini-VAC therapy) with the abdication of an open window thoracotomy (OWT) allows the fast and minimally invasive treatment of pleural empyema [5],[6].

In this report, we describe the novel application of Mini-VAC therapy in a conventional therapy resistant lung abscess.

## Methods

**Figure 1:**
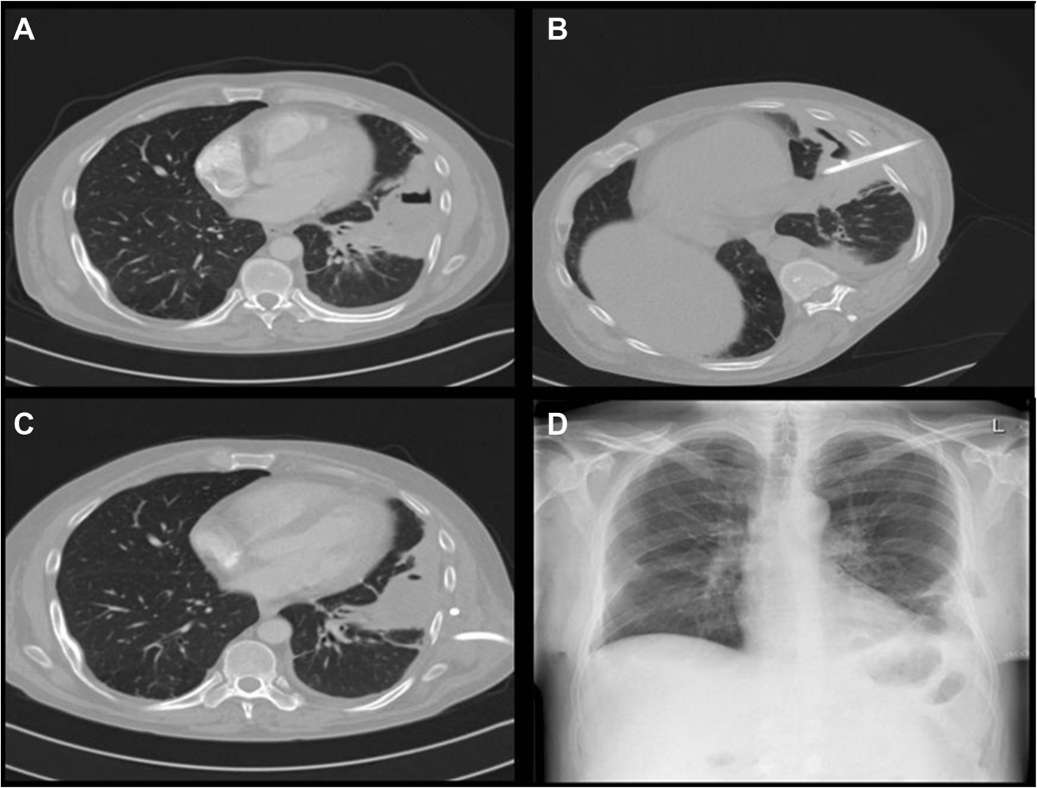
**Morphological features of the course of conservative therapy and follow-up. (A)** CT scan revealed a disintegrated mass of the left lower lobe. **(B)** Insertion of percutaneous catheter drainage. **(C)** Catheter dislocation. **(D)** Chest X-ray after VAC Therapy and before discharge.

**Figure 2:**
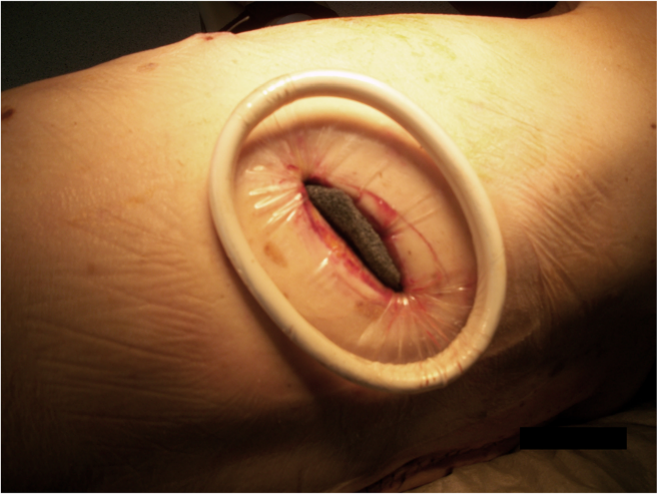
**End position of the ALEXIS wound protector/retractor and suction with 100 mm Hg on the vacuum-assisted closure (VAC) sponges.**

This investigation was approved by the local ethics committee of the Hospital Barmherzige Brüder (Ethikkomitee am Krankenhaus Barmherzige Brüder Regensburg). A 43-year-old man was diagnosed with an acute lung abscess in the left upper lobe. The patient abused alcohol and had a history of pneumonia with an intermittent febrile course. The production of purulent sputum became common within the last weeks/months. Microbiology of the sputum showed a mixture of *Prevotella buccae* and *Bacteroides* spp. The patient was started on intravenous piperacillin/tazobactam while awaiting sensitivity results. Fiberoptic bronchoscopy excluded significant airway obstruction and there was no visible draining bronchus of the abscess. Chest computed tomography (CT) showed a 5 to 7 cm disintegrated mass with air bubbles in the anterobasal and laterobasal segments of the left lower lobe (Figure 1). The abscess sat quite close to the visceral pleural surface. Because the missing of the transbronchial clearance it was a percutaneous catheter drainage (14 French) inserted under CT control (Figure 1). A whitish-yellow substance was aspirated, and the pH of the putrid contain of abscess was acidic (pH = 6.8). Seven days after this intervention, the clinically course showed no improvement and the patient had high elevated levels of C-reactive protein. Chest CT showed catheter dislocation (Figure 1) and no reduction in abscess size. The patient was planned for surgical intervention because he was septic. In consideration of the poor general condition of the patient (Karnofsky-Index = 50%), the decision was made to proceed with Mini-VAC therapy, including a soft tissue retractor (ALEXIS® retractor; Applied Medical, Rancho Santa Margarita, CA, U.S.A.) (Figure 2). Under general anesthesia, a 6 cm-long incision centered under CT guidance over the area of the lung abscess was performed. The intercostal muscle was divided, and the abscess cavity was opened. We created a circumscribed and open "empyema" cavity in terms of an abscess - pleural space - chest wall - skin communication. The surrounding lung was adherent to the parietal pleura, creating an isolation barriére.

Then, the ALEXIS® retractor was positioned. After intercostal aspiration of all pus and necrotic debris, the cavity was flushed with LAVANID 0.02% (Serag-Wiessner KG, Naila, Germany). VAC sponges (GranuFoam Dressings (KCI Medical, Wiesbaden, Germany), 400 - 600 microns) were inserted in the abscess cavity through the Alexis® retractor to fill the entire intrapulmonary space (Figure 2). The level of suction was initially set to 100 mm Hg, with a maximum suction of 125 mm Hg. The sponge was changed once in the operating room on the 3rd postoperative day, at which point the ALEXIS® retractor was changed as well. Incentive spirometry was part of therapy to facilitate remaining lung expansion.

## Results

After nine days of Mini-VAC treatment, the abscess disappeared. The remaining space was filled with Genta-Coll® resorb (Resorba GmbH, Nürnberg, Germany) - a hemostyptic gentamicin collagen sponge. The abscess cavity was sterile, and the mini-thoracotomy could be closed on the 10th postoperative day. The patient was discharged on the 11th postoperative day without any further complications. No recurrence occurred during long-term follow-up (13 months).

## Discussion

A lung abscess is a thick-walled cavity that contains purulent material and can occur at any age [7]. Approximately 90% of patients with lung abscesses can be cured by antibiotic therapy and/or thoracocentesis [8],[9]. The roles of surgery include the prevention of sepsis and managing complications. Complicated lung abscesses are associated with high mortality rates of up to 23% [10]. Standard surgical treatment includes debridement of the abscess or even pulmonary wedge/anatomical resection and is often combined with prolonged hospitalization [10].

Here, we demonstrated the application of Mini-VAC therapy in a debilitated patient with a complicated lung abscess for the first time.

The application of plastic wound retractors (ALEXIS® retractor) in managing pleural empyemas with Mini-VAC therapy is well described [3]-[6]. The same technique can also be easily used for the treatment of lung abscesses. An important advantage of plastic wound retractors use is that they act also as a barrier that protects the soft tissue and wound from bacterial translocation.

The first installation, and perhaps the first change of the VAC system, should be performed under general anesthesia. Further changes may be performed bedside.

Because a lung abscess is covered by granulation tissue during the healing of the surrounding parenchyma, no fistula in the lung parenchyma was detected, and the level of suction was steady. Secretions were collected in the VAC device. The continuous removal of potentially infectious secretions from the wound supported rapid healing of the initially septic condition.

Daily cleaning or dressing was not necessary, and we changed the VAC sponge only once over the course of 7 days. This procedure was very convenient for both the patient and the doctors.

Outpatient service with initial daily wound care performed by specialized nurse technicians would be advised.

The abscess cavity was rapidly cleaned and decreased in size. Closure of the chest was easy, without any short- or long-term treatment failure.

## Conclusions

The use of the Alexis® retractor and Mini-VAC therapy for a septic patient with lung abscess is a novel extension of use of negative pressure wound therapy in thoracic surgery. VAC systems should be considered for widespread use as an alternative option for the treatment of complicated pulmonary abscesses in elderly, debilitated, and immunocompromised patients after failed conservative treatment, but only if the abscess is located quite close to the visceral pleural surface. It is difficult to achieve lung abscesses localized paravertebral, paramediastinal or apical.

### Consent

Written informed consent was obtained from the patient for publication of this report and any accompanying images.

## Authors’ original submitted files for images

Below are the links to the authors’ original submitted files for images. Authors’ original file for figure 1 Authors’ original file for figure 2

## Abbreviations

VAC therapy: Vacuum-assisted closure therapy
Mini-VAC therapy: Minimally invasive vacuum-assisted closure therapy
CT: Computed tomography
OWT: Open window thoracotomy
