# Supplementary figures and images for: Vacuum-assisted closure therapy in the management of lung abscess
Source: J Cardiothorac Surg. 2014 Sep 6;9:157. doi: 10.1186/s13019-014-0157-x (PMC4172792; doi:10.1186/s13019-014-0157-x)

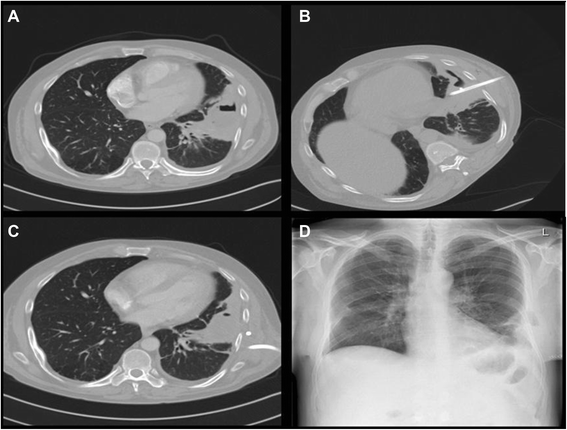

Supplement: Supplementary file 1 — Authors’ original file for figure 1 [file 13019_2014_157_MOESM1_ESM.gif]

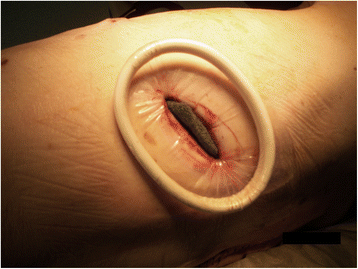

Supplement: Supplementary file 2 — Authors’ original file for figure 2 [file 13019_2014_157_MOESM2_ESM.gif]
